# Evaluation of fecal culture and fecal RT-PCR to detect *Mycobacterium avium* ssp. *paratuberculosis* fecal shedding in dairy goats and dairy sheep using latent class Bayesian modeling

**DOI:** 10.1186/s12917-016-0814-5

**Published:** 2016-09-20

**Authors:** Cathy A. Bauman, Andria Jones-Bitton, Jocelyn Jansen, David Kelton, Paula Menzies

**Affiliations:** 1 Department of Population Medicine, University of Guelph, Guelph, Ontario N1G 2W1 Canada; 2 Veterinary Science & Policy Unit, Animal Health and Welfare Branch, Ontario Ministry of Agriculture, Food & Rural Affairs, Elora, Ontario N0B 1S0 Canada

**Keywords:** Dairy goats, Dairy goats, Fecal PCR, Fecal culture, Paratuberculosis

## Abstract

**Background:**

The study’s objective was to evaluate the ability of fecal culture (FCUL) and fecal PCR (FPCR) to identify dairy goat and dairy sheep shedding *Mycobacterium avium* ssp. *paratuberculosis*. A cross-sectional study of the small ruminant populations was performed in Ontario, Canada between October 2010 and August 2011. Twenty-nine dairy goat herds and 21 dairy sheep flocks were visited, and 20 lactating females > two years of age were randomly selected from each farm resulting in 580 goats and 397 sheep participating in the study. Feces were collected per rectum and cultured using the BD BACTEC™ MGIT™ 960 system using a standard (49 days) and an extended (240 days) incubation time, and underwent RT-PCR based on the *hsp-*X gene (Tetracore®). Statistical analysis was performed using a 2-test latent class Bayesian hierarchical model for each species fitted in WinBUGS.

**Results:**

Extending the fecal culture incubation time statistically improved FCUL sensitivity from 23.1 % (95 % PI: 15.9-34.1) to 42.7 % (95 % PI: 33.0-54.5) in dairy goats and from 5.8 % (95 % PI: 2.3-12.4) to 19.0 % (95 % PI: 11.9-28.9) in dairy sheep. FPCR demonstrated statistically higher sensitivity than FCUL (49 day incubation) with a sensitivity of 31.9 % (95 % PI: 22.4-43.1) in goats and 42.6 % (95 % PI: 28.8-63.3) in sheep.

**Conclusions:**

Fecal culture demonstrates such low sensitivity at the standard incubation time it cannot be recommended as a screening test to detect shedding of MAP in either goats or sheep. Extending the incubation time resulted in improved sensitivity; however, it is still disappointingly low for screening purposes. Fecal PCR should be the screening test of choice in both species; however, it is important to recognize that control programs should not be based on testing alone when they demonstrate such low sensitivity.

**Electronic supplementary material:**

The online version of this article (doi:10.1186/s12917-016-0814-5) contains supplementary material, which is available to authorized users.

## Background

In Canada, the province of Ontario has the largest dairy small ruminant industries in the country. Over the last 10 years the industry has grown rapidly, but has been impacted by production-limiting diseases such as paratuberculosis, similar in scope to other dairy sectors. The true herd- and flock-level prevalences of paratuberculosis have been estimated to be 83.0 % (95 % PI: 62.6-98.1) in dairy goats and 66.8 % (95 % PI: 41.6-91.4) in dairy sheep [1]. This widely-recognized chronic enteric wasting disease of domestic ruminants is caused by infection with *Mycobacterium avium* ssp. *paratuberculosis* (MAP). Transmission is mainly fecal-oral, although infectious animals can also shed bacteria in colostrum and milk, and in utero transmission has been shown to occur [2]. Infectious animals are the primary source of infection as this facultative intracellular bacterium has not demonstrated the ability to replicate in the environment [3]. Detection and culling of infectious animals in the early stages of infection is one component of control programs in countries such as Canada where the paratuberculosis vaccine has not been approved for use. The presence of tuberculosis in North America means there is an inherent concern that vaccinated animals could not be differentiated from animals exposed to tuberculosis.

The general consensus is that fecal tests such as fecal culture (FCUL) and PCR are the most sensitive MAP tests and able to detect shedding animals first [4]. While the strength of fecal culture is that it consistently demonstrates almost perfect specificity [4], sensitivity estimates vary widely and are dependent on the stage of infection in the study population and on the MAP strain circulating in a given geographic region and animal species [5]. The S-strain (sheep strain) of MAP has been previously shown to be more sensitive to particular decontamination antibiotics and detergents [6], more fastidious about the components of the culture media it is grown in [6, 7], and takes longer to grow in vitro [8]. The current recommendation for incubating fecal samples from a previously untested population where the circulating strain is unknown is at least 8 months [7]. Since automated fecal culture systems often incubate samples for less than 60 days, the sensitivity of fecal culture may be underestimated in populations where the S-strain predominates.

In theory, fecal PCR performance should not be influenced by MAP strain and has the potential to demonstrate higher sensitivity [9, 10]. However, the test can exhibit lower than expected specificity if the specific gene sequence cross-reacts with other pathogens [11].

Neither fecal culture nor fecal PCR (FPCR) is a suitable MAP reference test with which to evaluate the other test. In the absence of a gold standard, test evaluations often use a latent class (LC) approach where no test is considered the gold standard and both tests are interpreted in parallel [12]. One LC model commonly in use is the Hui-Walter (HW) 2-test maximum likelihood estimates model. However, in order for this HW model to be identifiable, wherein there is sufficient degrees of freedom in the data to calculate the parameters of interest, it must fulfill three assumptions: the tests must be conditionally independent, the tested animals must be divided into two populations with different prevalences, and the two tests must demonstrate the same test sensitivity and specificity in each of the two populations [13, 14]. Fulfilling this last assumption is an inherent challenge with paratuberculosis tests as it is generally acknowledged that they demonstrate higher sensitivity in populations with higher prevalences [5, 15]. An alternative 2-test method is to use a hierarchical approach incorporating prior information in a Bayesian framework [16]. The main assumptions with this model are that prevalences may differ within subpopulations or herds, but that there is a fixed test sensitivity and specificity in the population analyzed as one group, the population is obtained randomly, and the animals tested represent the population or field conditions in which the test will be utilized [16].

As yet no MAP test evaluations have been undertaken in either of the dairy goat or dairy sheep populations in Canada. Therefore, the objective of this study was to evaluate the performance of fecal culture and fecal PCR. To account for the potential presence of the slow-growing S-strain of MAP, an additional objective was to assess the impact of extending the culture incubation time on the performance of the same 2 tests.

## Methods

### Herd and animal selection

This study was part of a larger cross-sectional study conducted between October 2010 and August 2011. The purpose of the original study was to determine prevalence, identify the risk factors for herds/flocks to test positive, and to evaluate the performance of 7 individual- and herd-level diagnostic tests. Greater detail on herd and animal sampling are available [1].

Briefly, 29 dairy goat herds and 21 sheep flocks in the province of Ontario were visited once during milking time, at which time 20 lactating does or ewes over the age of 2 years were selected as they came through the milking parlour using a formal systematic random sampling procedure. The inclusion criteria at the animal-level were: milking doe/ewe greater than 2 years of age and greater than 3 days post-kidding/lambing. Animals were excluded if no feces could be detected intra-rectum at the time of sampling; in such cases, the next animal fulfilling the inclusion requirements was substituted.

No farm had concurrent or recent cohabitation with other domestic ruminant species, and no farm had ever used a vaccine for the prevention of paratuberculosis in their animals. MAP-infection status on all farms was unknown to the researchers at the time of enrolment in the study.

### Sample collection and handling

A minimum of 20 g of feces was collected by the same handler from the rectum of each selected animal, and placed into a sterile plastic vial (1 per animal). Single-use polyethylene gloves lubricated with 0.3mLs of sterile lubricant (Lubricating Jelly, Healthcare Plus, Canadian Packaging Company, Toronto, CA) were changed between each animal to reduce risk of cross-contamination. Fecal samples were transported in a Styrofoam cooler with ice packs, and stored at 4-8 °C, until submission to the Animal Health Laboratory (AHL, Laboratory Services Division, University of Guelph, Guelph, ON) within 12-18 h of collection. Two tests were performed: FCUL and FPCR. Generally, feces were processed at the AHL while fresh; however, samples from 7 goat and 10 sheep farms were frozen at -80 °C due to high work-volume at the laboratory, and were processed within 7-21 days.

### Laboratory testing

All technicians processing samples did not have access to the results of the other fecal test.

### Culture of Mycobacterium avium ssp. paratuberculosis

Fecal samples underwent a 3-day decontamination procedure prior to being cultured using the liquid culture BD BACTEC™ MGIT™ 960 paraTB non-radiometric automated system (Becton Dickinson and Company, Franklin Lakes, NJ, US). In brief, 2.0 g of feces were mechanically broken up with a tongue depressor and vortexed with sterile, purified water prior to incubation with Bacto yeast extract and sodium pyruvate. After overnight incubation with 0.9 % hexadecylpyridinium chloride (HPC), the samples were decanted and incubated a second night with an antibiotic brew of amphotericin B, vancomycin HCl, and nalidixic acid sodium salt. On the third day, 0.1 mL of sample was inoculated into a BACTEC™ MGIT™ ParaTB medium tube supplemented with BACTEC™ MGIT™ ParaTB supplement (casein, catalase, oleic acid and bovine albumin), antibiotic brew and egg yolk. Each 7 mL tube of medium contains modified Middlebrook 7H9 Broth base with mycobactin J and a fluorescent indicator compound embedded in silicone located in the bottom. Also present in the broth base is dissolved oxygen that upon being consumed by multiplying organisms causes the indicator to fluoresce. The system monitors the samples every 60 min for signs of fluorescence. The standard incubation length for samples in the system is 49 days; however, this was extended to 240 days in order to give the opportunity for slower-growing strains of MAP (S-strain), which are more common in sheep than other domestic ruminants [17], to be detected.

The time in days that each sample took to fluoresce positive was recorded. Fluorescence positive colonies underwent acid-fast staining and PCR confirmation with the *hsp*X gene (Culture Confirmation Protocol, MAP Extraction System, Tetracore®, Rockville, MD, US) according to the manufacturer’s instructions. Briefly, 1 mL of broth was placed in a tube with glass beads and beadbeated for 5 min at 4800 rpm, centrifuged for 10 min at 16 000 × g and the supernatant processed using the VetAlert™ Johne’s Real-Time PCR (Tetracore®) according to manufacturer's instructions with modifications for the RocheLightCycler® 2.0 thermocycler (Roche Applied Science, Laval, QC). The cycling program of this thermocycler is 10s @ 95 °C for enzyme activation and a 2-step PCR for 50 cycles (95 °C × 5 s and 95 °C x 30s). Samples that reached fluorescence with a cycle count (Ct) ≤ 42.0 were considered positive. Therefore, for a fecal sample to be designated culture positive it had to fluoresce, have a positive PCR confirmation test, and stain positive using acid-fast stain.

### Direct real-time polymerase chain reaction test (RT-PCR)

Prior to the fecal culture decontamination procedure feces underwent DNA extraction using the Two Gram MAP Extraction and Mini Beadbeater protocols (Tetracore®) according to manufacturer’s instructions. In summary, 2.0 g of feces were manually broken apart as described above, vortexed with 35 mL of DNase free water in a conical tube, and then allowed to incubate at room temperature for 30 min to allow the settling of larger particulate matter. The top 20 mL were removed and centrifuged in a new conical tube at 2500 × g for 10 min after which the pellet was removed and re-suspended in 1 mL of 1 × TE (Tris-HCl/EDTA) and placed in a disruption tube containing glass beads. The sample was briefly vortexed and then beadbeated at 4800 rpm for 5 min. DNA purification occurred according to manufacturer’s instructions and the sample underwent PCR using the VetAlert™ Johne’s Real-Time PCR (Tetracore®) with modifications for the RocheLightCycler® 2.0 thermocycler (Roche Applied Science, Laval, QC). Samples that reached fluorescence with a cycle count (Ct) ≤ 42.0 were considered positive.

Even though the *hsp*X gene is thought to be exclusive to MAP [18], there was a concern regarding possible cross-reaction with antigens of *Corynebacterium pseudotuberculosis*, the causal agent of caseous lymphadenitis (CL), a common disease in Ontario sheep and goats. Antibodies to *C. pseudotuberculosis* had earlier been shown to cause false positive reactions in a previously available ELISA [19] and no literature could be found indicating *hsp*X had been evaluated against *C. pseudotuberculosis* antigens. Therefore, ten samples of *C. pseudotuberculosis* from a stock of pure culture obtained from the AHL were processed as described above and evaluated using the same real-time PCR test.

### Purpose, target conditions, and case definitions

The purpose of this evaluation was to provide an accurate appraisal of test sensitivities and specificities for both fecal tests to evaluate if fecal PCR could be substituted for fecal culture in future test evaluations and to see if these results would be impacted by extending the culture period. The target condition for this test evaluation was MAP infectious animals which as a case definition, was animals sufficiently infected with MAP that they shed enough bacteria in their feces to potentially test positive on fecal culture, fecal PCR or both.

A ‘contaminated’ fecal sample was defined as a sample that signalled positive in the MGIT fecal culture system by demonstrating fluorescence, but was negative on one or more of the confirmation tests (acid-fast and/or PCR).

### Statistical analyses

All data were entered and managed in Microsoft® Excel (2007). Descriptive statistics and contingency tables were generated in StataIC® 11.1 (StataCorp LP, College Station, TX, US). The tests were evaluated separately in goats and sheep as it was expected that there would be species-specific differences in test performance [20]. Test sensitivities and specificities were determined using a 2-test multiple populations latent class Bayesian model with a random effect as described previously [16, 21]. This model allowed for dependence between the 2 fecal tests [22] as they are based on a similar biological process. Non-informative priors were used for test sensitivity. Specificity priors were based on previously published literature [4, 19, 22, 23, 24] that evaluated tests in sheep and goats when available and other species when not. These priors were reviewed for appropriateness by a researcher with small ruminant MAP experience (MT Collins, University of Madison, WI). The final priors were represented by beta distributions in the format (a,b) and generated using the software BetaBuster (http://betabuster.software.informer.com). The same specificity priors were used for both the goat and sheep models. Priors for the prevalence parameters were based on the data acquired during an earlier study [1]. Separate herd-level (HLP) and within-herd prevalence (WiHP) priors were used for each species. The herd-level prior was represented by a beta distribution (also generated through BetaBuster) while the prior for the estimated prevalence within an infected herd was represented by a normal distribution in the format (μ, precision of μ). The ‘true’ within-herd prevalence of any sampled herd or flock was represented by a zero-inflated logit-normal variable that was a function of the probability of the herd/flock to be infected (FP), the WiHP given the farm was infected and a random effect (U). The random effect represented the potential variability in prevalence between farms and was modeled by a normal distribution with a mean of 0 and a precision (prec) represented by a non-informative gamma distribution [21]. All prior data is available in Table 1.

**Table 1:** Prior information used in the 2-test latent class Bayesian hierarchical model developed to estimate test sensitivity and specificity for 2 paratuberculosis fecal tests in dairy goats and dairy sheep in Ontario, Canada

Parameter	Distribution	Dairy Goats			Dairy Sheep		
		(a, b)	95 % certain	Mode	(a, b)	95 % certain	Mode
Fecal culture specificity	Beta	560.72, 6.65	> 0.98	0.99	560.72, 6.65	> 0.98	0.99
Fecal PCR specificity	Beta	99.70, 6.19	> 0.90	0.95	99.70, 6.19	> 0.90	0.95
Herd-level prevalence	Beta	19.48, 5.62	> 0.63	0.80	8.35, 4.62	> 0.42	0.67
		precision μ	95 % certain	Median	precision μ	95 % certain	Median
Within-herd prevalence	Normal	175.41	< 0.50	0.35	55.62	< 0.74	0.48

The sheep and goat models were each fitted using WinBUGS software (Available at: http://www.mrc-bsu.cam.ac.uk/software/bugs/the-bugs-project-winbugs/). Posterior estimates for test sensitivity and specificity were generated using the Markov Chain Monte Carlo (MCMC) sampling method and the Gibbs algorithm. Model convergence was assessed visually by examining the traces, histories, Monte Carlo (MC) errors and autocorrelations plots. Diagnostically, CODA outputs were generated from WinBUGS and evaluated using the coda package in [25] (R) and the Raftery-Lewis diagnostic [26]. In this package the I statistic is examined for variations exceeding 5 and the M (minimum burnin period) and N (minimum iterations needed) are checked for values exceeding those used in the primary model [27]. To assess the influence of the initial values, the Gelman-Rubin [28] statistic in WinBUGS was generated using 3 different sets of starting points. To assess the need to account for dependence between the 2 fecal tests 4 models were run: fully independent, fully dependent, 1 with only a covariance in the diseased state and 1 with only the covariance on the non-diseased state. After each model was run, any covariances with a 95 % probability interval that contained zero were removed and the model was re-run.

The final estimates of the test and prevalence parameters generated from the model were represented by the median and its 95 % probability intervals (PI) after the first 10 000 iterations were dropped as the burnin period and 60 000 iterations were run. To determine the probability of one test demonstrating a statistically important difference from another test the Bayesian posterior probability (POPR) [29] was estimated. The POPR is calculated by first creating a Boolean or indicator variable using the step function in WinBUGS and a non-specific node X [30]. The value of 1 is then assigned to step(X) when the function is ‘true’ (e.g. Se FPCR- Se FCUL ≥ 0) and the value of 0 when the function is ‘false’ (e.g. Se FPCR- Se FCUL < 0) thus acting as a counter of the number of iterations where this statement is true. The mean value of X subsequently represents the Monte-Carlo estimate of the posterior probability that there was a statistically important difference between the 2 tests; a POPR threshold of ≥0.95 was used as the cut-off [20].

Sensitivity analysis was performed to assess the influence of the priors on the sensitivity and specificity estimates. A table of the values uses in this analysis is available in the Additional file 1. The model was also run using 3 different chains of starting points to assess the influence that these points had on the model results and assessing the Gelman-Rubin statistic [28].

## Results

### Test population

Fecal samples were collected on 29 dairy goat farms from 580 dairy goats representing Saanen, Alpine, Toggenberg, Nubian, La Mancha breeds, and their crosses. Fecal samples were also collected on 21 dairy sheep farms from 400 dairy sheep representing East Friesian, British Milk Sheep breeds and their crosses. However, samples from 3 sheep were later discarded, as they were determined to be less than 2 years of age resulting in a sample size of 397 ewes.

### Laboratory results

#### Culture of Mycobacterium avium ssp. paratuberculosis

When the standard incubation time (49 days) was used, the proportion of fecal positive dairy goats was 9.8 % (57/580) and fecal positive dairy sheep was 2.8 % (11/397) (Tables 2 and 3 respectively).

**Table 2:** Comparison of *Mycobacterium avium* ssp. *paratuberculosis* fecal shedding status as determined by fecal culture using standard and extended incubation times, and fecal PCR, in dairy goats sampled in Ontario, Canada in 2010–2011

		Fecal PCR positive	Fecal PCR negative	Total
Standard Incubation Time (49 days)	Fecal culture positive	30 (5.2 %)	54 (9.3 %)	57 (9.8 %)
	Fecal culture negative	66 (11.4 %)	457 (78.8 %)	523 (90.2 %)
	Total	96 (16.6 %)	484 (83.4 %)	580
Extended Incubation Time (240 days)	Fecal culture positive	35 (6.0 %)	71 (12.2 %)	106 (18.3 %)
	Fecal culture negative	61 (10.5 %)	413 (71.2 %)	474 (81.7 %)
	Total	96 (16.6 %)	484 (83.4 %)	580

**Table 3:** Comparison of *Mycobacterium avium* ssp. *paratuberculosis* fecal shedding status as determined by fecal culture using standard and extended incubation times, and fecal PCR, in 397 dairy sheep sampled in Ontario, Canada in 2010–2011

		Fecal PCR positive	Fecal PCR negative	Total
Standard Incubation Time (49 days)	Fecal culture positive	4 (1.0 %)	7 (1.8 %)	11 (2.8 %)
	Fecal culture negative	56 (14.1 %)	330 (83.1 %)	386 (97.2 %)
	Total	60 (15.1 %)	337 (84.9 %)	397
Extended Incubation Time (240 days)	Fecal culture positive	10 (2.5 %)	20 (5.0 %)	30 (7.6 %)
	Fecal culture negative	50 (12.6 %)	317 (79.8 %)	367 (92.4 %)
	Total	60 (15.1 %)	337 (84.9 %)	397

The proportions of fecal samples that were positive after the extended incubation time (240 days) were 18.3 % (95 % PI: 15.1–21.4 %) in dairy goats and 7.6 % (95 % PI: 4.9-10.2 %) in dairy sheep. Using extended incubation, the mean times for fecal samples to culture positive (machine-signalled and confirmed by PCR and acid-fast staining) was 66.4 days (median: 36.0 days) for goat samples and 104.2 days (median: 54.5 days) for sheep samples.

The proportion of contaminated fecal samples at the 240-day cut-off was 41.6 % in goats (241/580) and 34.3 % (136/397) in sheep. Of these, 38/241 goats (15.8 %) and 21/136 sheep (15.4 %) samples were positive on the direct PCR test performed prior to the samples undergoing decontamination and culture. Contamination proportions were not significantly different between frozen and fresh samples (goats *χ*^2^ = 3.131, *p* < 0.0777; sheep *χ*^2^ = 0.1412, *p* < 0.7077).

#### Direct real-time polymerase chain reaction test (RT-PCR)

The proportion of FPCR-positive samples was 16.6 % (96/580) in dairy goats and 15.1 % (60/297) in dairy sheep. The Ct values for FPCR-positive samples ranged from 23.96–41.66 (mean positive Ct =36.04; median Ct =36.63) for goats and 21.88-41.19 (mean positive Ct =35.67; median Ct = 36.68) for sheep. FPCR was negative on all 10 samples of *C. pseudotuberculosis* tested.

### Test performance

#### *Dairy goat*s

Sensitivity and specificity results for both tests are presented in Table 4. Extending the culture period statistically increased the test sensitivity for FCUL from 23.1–42.7 % (*p* = 0.9954). Specificity was not statistically different (*p* = 0.5735) when estimated at the extended time. In addition, the sensitivity (*p* = 0.5754) and specificity (*p* = 0.4832) for FPCR estimated in each of the 2 different models did not demonstrate a statistically important difference from one another.

**Table 4:** Performance of two paratuberculosis tests (fecal culture and fecal PCR) at two different culture incubation times in 580 dairy goats in Ontario, Canada

	Standard Incubation Time		Extended Incubation Time	
	Fecal culture (95 % PI)	Fecal PCR (95 % PI)	Fecal culture (95 % PI)	Fecal PCR (95 % PI)
Sensitivity	23.1 % (15.9-34.1)	31.9 % (22.4-43.1)	42.7 % (33.0-54.5)	30.5 % (23.3-38.8)
Specificity	98.9 % (98.0-99.5)	93.3 % (89.0-97.1)	98.8 % (97.8-99.5)	93.4 % (90.0-96.5)

PI = probability interval

Sensitivity of fecal culture was not statistically lower (*p* = 0.9488) than fecal PCR when evaluated at the standard time, but was statistically higher (*p* = 0.9902) than the sensitivity of PCR at the extended time. At both the 49-day (*p* = 0.9996) and 240-day (*p* = 0.9998) incubation periods the specificity of fecal culture was statistically higher than the specificity of fecal PCR.

#### Dairy sheep

Test sensitivities and specificities determined using the standard and extended incubation time are listed in Table 5. Increasing the incubation time of cultured samples, statistically improved the sensitivity of FCUL from 6.0–19.7 % (*p* = 0.9944), but had no impact on specificity (*p* = 0.7762). FPCR test sensitivity (*p* = 0.7829) and specificity (*p* = 0.3359) were not statistically impacted by the extended incubation time. At the standard incubation time, FPCR was statistically more sensitive than FCUL (*p* = 1.0), but FCUL was statistically more specific than FPCR (*p* = 0.9967). At extended incubation times, the FPCR test was still statistically more sensitive than FCUL (*p* = 0.9940), and FCUL was still statistically more specific than FPCR (*p* = 0.9931).

**Table 5:** Performance of two paratuberculosis tests (fecal culture and fecal PCR) at two different culture incubation times in 397 dairy sheep in Ontario, Canada

	Standard Incubation Time		Extended Incubation Time	
	Fecal culture (95 % PI)	Fecal PCR (95 % PI)	Fecal culture (95 % PI)	Fecal PCR (95 % PI)
Sensitivity	5.8 % (2.3-12.4)	42.6 % (28.8-63.3)	19.0 % (11.9-28.9)	35.3 % (24.7-49.8)
Specificity	98.8 % (97.8-99.4)	97.8 % (93.0-99.9)	98.9 % (97.8-99.5)	97.4 % (93.5-99.7)

PI = probability interval

#### Latent class Bayesian models

Based on visual observation of the model traces, histories, and densities there was no evidence that convergence of the model had not occurred. The autocorrelations did not drop off to 0 before 10 on the sensitivity parameters therefore the model was thinned by a value of 10. This was further corroborated when the Raftery-Lewis diagnostic was performed in R and some of the I values were between 12–15 and decreased to less than 5 after thinning occurred. The starting values had little impact on the posterior estimates as the BGR diagnostic approached 1.0 for all parameters. Sensitivity analysis indicated that the specificity priors for FCUL and FPCR most influenced the goat models. When the prior for FCUL was made the same as the FPCR prior or lower, the sensitivity estimate for FCUL was increased to 22.5 % (standard time) and 43.8 % (extended time) in goats, with the other parameters remaining unchanged (<2 % change); the sheep estimates were unaffected. The specificity prior for fecal PCR only impacted the goat model results when it was increased to the same confidence level as FCUL. Again, the impact of increasing this prior was only on the sensitivity estimates for FCUL, increasing it to 21.1 % (standard time) and 36.3 % (extended time) in goats, but leaving all other parameters unchanged; again, increase of the prior did not affect the sheep results. All other priors had limited impact on the posterior estimates.

When assessing dependence of all models the only covariance interval that did not contain 0 was the covariance in the diseased state for the goat tests at the standard incubation time (covDFS). Therefore, covDFS was included in the primary analysis goat standard incubation model. All covariances evaluated in the sheep models contained 0, therefore the completely independent model was used for the primary analysis.

## Discussion

### Test population and study design

The objective of this study was to evaluate 2 paratuberculosis fecal tests to determine which test should be used for potentially screening dairy does and ewes for fecal shedding of MAP. To have external validity, reduce selection bias, and fulfill the model assumptions, the study population must be representative of the target population within which the tests will be applied and is best accomplished through formal random sampling at the herd and animal level [16, 31]. For all herds and animals in the dairy goat population this was successfully achieved; however, the ability to randomly sample the sheep flocks was hampered by the fact that dairy sheep are not licensed under the provincial Milk Act (Milk Act, 1990, Available at https://www.ontario.ca/laws/statute/90m12) and therefore, no complete sampling frame exists. Every attempt was made to contact producers from all the milk processors across Ontario and the participating farms represented a variety of flock sizes, regions, and management styles. In addition, random selection of ewes occurred on-farm, therefore the researchers involved are confident that the dairy sheep sample population is reflective of the target population. Random sampling also helps to minimize spectrum bias which occurs when animals from one stage of infection are more likely to be sampled than another stage. Since paratuberculosis tests demonstrate higher sensitivity in the later stages of infection it is important that the spectrum of infection in the study population match the range of infection stages in the target population. No pre-selection of animals based on weight, thriftiness or the presence/absence of diarrhea occurred so as to mimic the conditions experienced when screening animals.

### Sensitivity analysis of model

The high level of confidence placed on the specificity prior for FCUL had a large influence on the posterior estimates for the goat models; however, due to the large number of bacteria that need to be present in a sample to culture positive, and the double-confirmation step there is strong support for this prior to be as high as it is. The specificity prior for FPCR only impacted the goat estimates when it was increased to the same prior as FCUL. There is not enough evidence to date to warrant weighting the specificity prior of FPCR equal to FCUL. It is unclear at the moment why these priors had little impact on the sheep estimates. Otherwise, all the models converged well.

### Laboratory test performance

#### Fecal culture

Overall, the average length of time for cultures to signal positive in this test evaluation (goats: 66 days, sheep: 100 days) fell outside of the standard length of time that cultures would normally be kept (49 days). Thirty per cent of positive goat cultures and 60 % of positive sheep cultures would have been classified as negative had the standard time been observed. Increasing the incubation time of cultured samples dramatically improved test sensitivity of FCUL from 23.1 % (95 % PI: 15.9-34.1) to 42.7 % (95 % PI: 33.0-54.5) in the dairy goat population and from 5.8 % (95 % PI: 2.3-12.4) to 19.0 % (95 % PI: 11.9-28.9) in the dairy sheep population. Even though the sensitivity is statistically higher in both species, increasing incubation times would not be practical for screening animals in the general population. FCUL at the standard incubation time is already an expensive, labor-intensive procedure with an impractical delay between sample submission and acquisition of results for producers and veterinarians. However, extending the incubation time to 240 days should be considered in test evaluation and prevalence studies where the objective is to maximize the sensitivity of the test. Small ruminant prevalence studies are likely to be significantly under-estimating the level of infection when using FCUL at the standard time. It must be noted, that even if incubation time is extended, under-reporting would likely still occur as the fluorescent indicator present in the media has a limited life-expectancy. The actual life-expectancy has not been published, however there is the possibility that some of the samples in this study did not ‘signal’ positive, even with the extra incubation time, because the indicator was no longer active.

Fecal culture using the BACTEC™ MGIT™ system demonstrated such low sensitivity in sheep that it cannot be recommended in this species in this region for any purpose. Gumber and Whittington [24] previously expressed concern that the liquid culture media (modified Middlebrook 7H9) in this culture system may not support the growth of S-strains of MAP either due to the lower concentration of egg yolk than is present in other media or the presence of vancomycin in the antibiotic brew used to prevent contamination. While the S-strain has been most commonly detected in sheep in other countries [17, 32] it has also been isolated from cattle, goats and other species [8]. Conversely the other strains, C- (cattle) and I- (intermediate) strains, have also been detected in sheep [32]. Prior to this study, the only other previous literature available on the MAP strains present in Canadian sheep was a study in 1990 [32] that used hybridization and restriction analysis to identify strain types. Seven different Canadian sheep fecal/intestinal tissue samples were processed and identified as 6 cattle strains and 1 intermediate strain. However, given the current test results it is suspected that the S-strain may be the predominant strain in this population and requires further investigation.

#### Fecal PCR

The use of FPCR has the potential to be a rapid, relatively inexpensive, and sensitive method of MAP diagnosis, especially in sheep where FCUL performed so poorly. However, PCR-based tests are only as useful as the uniqueness of the DNA primers used. PCR MAP tests based on the IS900 gene sequence had previously cross-reacted with other Mycobacterium species [11, 33] and therefore, had limited usefulness. Evaluations of the Tetracore® *hsp*X sequence to date report no cross-reaction with common bacterial pathogens [18], (written communication, Tracy Fecteau, Tetracore®). However, prior to this study, the test had not been evaluated against *C. pseudotuberculosis* the causative agent of caseous lymphadenitis*.* Antibodies to this bacteria demonstrated cross-reactivity with a previous MAP ELISA test used in goats and sheep [19]. While antibody cross-reactivity does not necessarily infer DNA cross-reactivity, it was reassuring that the Tetracore® test did not identify any of the *C. pseudotuberculosis* samples as positive.

Overall, the performance of FPCR was disappointing in this study with a sensitivity of 30–40 %. While it performed significantly higher than FCUL (at the standard incubation time) in both species, previous literature had indicated a potential sensitivity of 60 % [23] could be achieved. The detection limit for the Tetracore® test, in theory, is a single gene from one bacterium, and much lower than the detection limit of FCUL (2.3×10^1^–2.3×10^3^ bacteria) [24] therefore the test should be able to detect animals shedding at a low level. However, it is difficult to isolate DNA from feces as they contain compounds that can inhibit the PCR reaction: bile salts, ionic detergents, hemoglobin degradation products, polyphenol-based substances, and humic-like acid [34]. Additionally, MAP aggregates in clusters [34], so even when a substantial amount of bacteria may be present in a sample it may be missed when only a 2.0 g amount is processed as the protocol requires. The Alinovi study likely demonstrated higher sensitivity because they studied cattle [23]. While the influence of animal species is not referred to in the manufacturer’s instructions for the Tetracore® kit, it specifically states that sampled feces should be fresh and moist. Small ruminant feces are inherently dry in comparison to cattle. Goat and sheep feces are excreted in formed, firm pellets that are difficult to manually break apart. This hampers the ability to properly prepare the sample for FPCR testing. Alinovi’s study also used culture-positive clinically affected animals. The cattle tested were shedding MAP bacteria at a high enough level to culture positive [23]; therefore, there would be more bacteria to potentially detect via PCR thus improving sensitivity. MAP tests typically exhibit lower test sensitivity when evaluated in populations of sub-clinically rather than clinically diseased animals [35]. In this study, where the MAP infection status was unknown, animals would potentially be in the early stages of infection, thereby shedding organisms at very low levels, which would reduce the perceived sensitivity, but better represent what is encountered when screening animals in the general population. Further work is needed to identify if the dry feces of small ruminants can be processed in a way to further improve the recovery of DNA and possibly improve sensitivity.

## Conclusions

Fecal culture is extremely useful in test comparison and prevalence studies as the 2-step confirmation process almost ensures 100 % specificity, and as demonstrated in this study, its sensitivity can be statistically increased by extending the incubation time. However, compared to FCUL, FPCR is more economical, has a shorter turnaround time, and is thus more likely to be accepted by veterinarians and producers. In this study, FPCR also demonstrated statistically higher sensitivity than FCUL (incubated at the standard time) in both species, and with a specificity exceeding 93 %, it should be the screening test of choice in both these species. However, a sensitivity of 30–40 %, means that for every animal identified as positive there are 2–3 animals undetected. As such, a successful paratuberculosis control program cannot be based solely on animal testing alone, and must incorporate other management and biosecurity measures.

## Additional file

Additional file 1: WinBUGS dairy goat model code. Bayesian latent class model used to generate the goat test results for this manuscript. (DOCX 13 kb)

## Abbreviations

MAP: Mycobacterium avium ssp. paratuberculosis
PI: Probability interval
