# Supplementary material for: Evaluation of fecal culture and fecal RT-PCR to detect Mycobacterium avium ssp. paratuberculosis fecal shedding in dairy goats and dairy sheep using latent class Bayesian modeling
Source: BMC Vet Res. 2016 Sep 20;12:212. doi: 10.1186/s12917-016-0814-5 (PMC5029031; doi:10.1186/s12917-016-0814-5)
Supplement: Additional file 1: — WinBUGS dairy goat model code. Bayesian latent class model used to generate the goat test results for this manuscript. (DOCX 13 kb) [file 12917_2016_814_MOESM1_ESM.docx]

**Dairy goat model**

model

{

# Extended Incubation time in goats

for (i in 1:29)

{

yE[i,1:KE, 1:KE] ~ dmulti(popE[i,1:KE, 1:KE], nE)

popE[i,1,1] <- pE[i]*(SeFPE*SeFCE) + (1-pE[i])*((1-SpFPE)*(1-SpFCE))

popE[i,1,2] <- pE[i]*(SeFPE*(1-SeFCE)) + (1-pE[i])*((1-SpFPE)*SpFCE)

popE[i,2,1] <- pE[i]*((1-SeFPE)*SeFCE) + (1-pE[i])*(SpFPE*(1-SpFCE))

popE[i,2,2] <- pE[i]*((1-SeFPE)*(1-SeFCE)) + (1-pE[i])*(SpFPE*SpFCE)

pE[i] <- FPE[i] * TWHPE[i]

## Is farm positive

FPE[i] ~ dbern(HLPE)

logit(TWHPE[i]) <- IWiHPE + UE[i]

UE[i] ~ dnorm(0, precE)

}

## Standard Incubation time

for (i in 1:29)

{

yS[i,1:KS, 1:KS] ~ dmulti(popS[i,1:KS, 1:KS], nS)

popS[i,1,1] <- pS[i]*(SeFPS*SeFCS+covDFS) + (1-pS[i])*((1-SpFPS)*(1-SpFCS))

popS[i,1,2] <- pS[i]*(SeFPS*(1-SeFCS)-covDFS) + (1-pS[i])*((1-SpFPS)*SpFCS)

popS[i,2,1] <- pS[i]*((1-SeFPS)*SeFCS-covDFS) + (1-pS[i])*(SpFPS*(1-SpFCS))

popS[i,2,2] <- pS[i]*((1-SeFPS)*(1-SeFCS)+covDFS) + (1-pS[i])*(SpFPS*SpFCS)

pS[i] <- FPS[i] * TWHPS[i]

## Is farm positive

FPS[i] ~ dbern(HLPS)

logit(TWHPS[i]) <- IWiHPS + US[i]

US[i] ~ dnorm(0, precS)

}

LLDFS <- (SeFCS-1)*(1-SeFPS)

ULDFS <- min(SeFCS,SeFPS) - SeFCS*SeFPS

covDFS ~ dunif(LLDFS, ULDFS)

rhoDFS<- covDFS / sqrt(SeFCS*(1-SeFCS)*SeFPS*(1-SeFPS))

## Priors

# Sensitivity and specificity

SeFCS ~ dbeta(1,1) ## Non-informative

SeFCE ~ dbeta(1,1) ## Non-informative

SpFCS ~ dbeta(560.72, 6.65) ## Mode=0.99, 95% sure >0.98

SpFCE ~dbeta(560.72, 6.65) ## Mode=0.99, 95% sure >0.98

SeFPS ~ dbeta(1,1) ## Non-informative

SeFPE ~ dbeta(1,1) ## Non-informative

SpFPS ~ dbeta(99.7,6.19) ## Mode=0.95, 95% sure >0.9

SpFPE ~ dbeta(99.7,6.19) ## Mode=0.95, 95% sure >0.9

#Infected herds - within prevalence

IWiHPE ~ dnorm(0.35, 175.41) ## Median=0.35, 95% sure <0.498

IWiHPS ~ dnorm(0.35, 175.41) ## Median=0.35, 95% sure <0.498

# precision (1/variance) of within-herd prevalence (alpha)

precS ~ dgamma(0.001, 0.001)

precE ~ dgamma(0.001, 0.001)

#proportion of infected herds

HLPS ~ dbeta(19.48,5.62)## Mode=0.8, 95% sure > 0.63

HLPE ~ dbeta(19.48, 5.62)## Mode=0.8, 95% sure > 0.63

pdSeF<-SeFCE-SeFPE

sdSeF<-step(pdSeF)

pdSeFS<-SeFPS-SeFCS

sdSeFS<-step(pdSeFS)

pdSpF<-SpFCE-SpFPE

sdSpF<-step(pdSpF)

pdSpFS<-SpFCS-SpFPS

sdSpFS<-step(pdSpFS)

pdSeFC<-SeFCE-SeFCS

sdSeFC<-step(pdSeFC)

pdSpFC<-SpFCS-SpFCE

sdSpFC<-step(pdSpFC)

pdSeFP<-SeFPS-SeFPE

sdSeFP<-step(pdSeFP)

pdSpFP<-SpFPS-SpFPE

sdSpFP<-step(pdSpFP)
